# Effectiveness of Early Rehabilitation with Exergaming in Virtual Reality on Gait in Patients after Total Knee Replacement

**DOI:** 10.3390/jcm11174950

**Published:** 2022-08-23

**Authors:** Anna Hadamus, Michalina Błażkiewicz, Kamil T. Wydra, Aleksandra J. Kowalska, Małgorzata Łukowicz, Dariusz Białoszewski, Wojciech Marczyński

**Affiliations:** 1 Department of Rehabilitation, Faculty of Medical Sciences, Medical University of Warsaw, 02-091 Warsaw, Poland; 2 Faculty of Rehabilitation, The Józef Piłsudski University of Physical Education in Warsaw, 00-809 Warsaw, Poland; 3 Professor Adam Gruca Independent Public Teaching Hospital in Otwock, Rehabilitation Clinic, 05-400 Otwock, Poland; 4 Medical Centre for Postgraduate Education, 01-813 Warsaw, Poland

**Keywords:** arthroplasty, gait analysis, games, knee, osteoarthritis, pressure mapping, spatiotemporal parameters, virtual reality

## Abstract

Total knee replacement (TKR) is the treatment of choice for advanced stages of osteoarthritis but it requires good postoperative rehabilitation. This study aimed to assess the effectiveness of exercises using virtual reality to improve gait parameters in patients after TKR. Fifty-nine patients 7–14 days after TKR surgery were divided into a study group (VRG, *n* = 38) and a control group (CG, *n* = 21). Both groups underwent the same 4-week rehabilitation protocol. The VRG group had 12 additional nonimmersive virtual reality game sessions on the Virtual Balance Clinic prototype system at 30 min each, focusing on gait and balance improvement. Spatiotemporal, force and foot plantar pressure parameters were collected on an instrumented treadmill during a 30 s walk. The most significant improvement was in the symmetry indices of forefoot force, maximum forefoot force, loading response time, and preswing time (*p* < 0.05) in both groups. Gait speed increased by 31.25% and 44% in the VRG and CG groups, respectively (*p* < 0.005). However, the extra exergaming sessions did not significantly improve rehabilitation outcomes. Therefore, additional VR training does not improve gait better than standard rehabilitation alone, but the improvement of gait, especially its symmetry, is significant within the first six weeks after surgery.

## 1. Introduction

Primary osteoarthritis (OA) in the knee joint is a chronic disease that mainly affects elderly individuals. It causes several problems: from pain and dysfunction in activities of daily living to psychological distress [1]. Patients with OA demonstrate a significant decrease in proprioception [2], which directly affects body balance and gait, leading to poorer mobility and a lower quality of life. Gait deviations in patients with advanced OA mostly include asymmetries in single support time (limping) [3], reduced knee range of movement (ROM), a loss of the biphasic nature of gait, and a reduced loading rate [4].

The OA treatment strategy includes several invasive and noninvasive procedures, which are selected based on the stage of the disease. In advanced stages, total knee replacement (TKR) is an invasive procedure that allows the patient to restore mobility [5]. The main goals of early rehabilitation after TKR are pain reduction and improving function, balance, and gait [6,7] to function independently in daily living. The gait abnormalities observed in patients after TKR are asymmetry in step length, single support time [3], and weight-bearing [7], and they are similar to those observed in knee-OA patients [4]. Previous studies reported that aquatic therapy, ergometer cycling, and intensified exercise programs improved selected gait parameters [6]. Gait training is also a part of the rehabilitation protocol in many clinical centres and is implemented to maximize outcomes [8,9].

In the recent years, exergaming in immersive, semi-immersive, or nonimmersive virtual reality (VR) increased in popularity. Its advantages include, among others, greater patient motivation, improved (faster) effects of training, and the possibility of correcting movement strategies using feedback features of games [10,11,12]. A growing number of studies have shown the utility of training with virtual reality in restoring gait and balance in older people, especially in the backwards stepping test, crossover stepping test [13], 6-min walking test [14], and timed up-and-go test [15]. This finding suggests that including VR games dedicated to improving balance and gait function in physiotherapy could increase the effectiveness of rehabilitation in patients after lower limb surgery such as TKR. However, according to Gumaa and Rehan [16], the outcomes of specialized VR and conventional treatment did not significantly differ in OA patients, TKR patients, and patients with other orthopedic conditions. Phu et al. [15] also confirmed this fact in a group of older adults. Conversely, Gazendam et al. [17] pointed out that VR training can be beneficial for some patients after TKR.

Previous studies primarily examined the utility of VR exergaming in neurological disorders [10,12,18,19] or elderly individuals [13,14,15,20] and mostly included balance outcomes. Few studies have examined the effectiveness of rehabilitation with VR on functional performances in patients after total knee replacements [16,17,21,22,23], and their results do not confirm that VR games are beneficial in this group of patients. Only a few publications concerning the effectiveness of VR intervention assessed selected gait parameters, mainly gait speed. We found no study assessing pressure, force, and spatiotemporal gait parameters in post-TKR patients who received virtual reality training. Therefore, this study aimed to evaluate the effectiveness of a standard rehabilitation program compared to the standard rehabilitation program with additional exercises in nonimmersive virtual reality in improving spatiotemporal and pressure distribution gait parameters in patients after total knee arthroplasty. Patients that had additional exergaming sessions in VR focused on gait and balance exercises were hypothesized to achieve improved gait results than the control group.

## 2. Materials and Methods

### 2.1. Participants

Fifty-nine patients 7–14 days after total knee replacement surgery were enrolled in the study. All patients were operated on at the Orthopedic Department of the “Professor Adam Gruca Independent Public Teaching Hospital” in Otwock, Poland. The sample size was calculated according to spatiotemporal parameter data available in the literature at the moment the project started. The required number of patients in each group was *n* = 20 for a *t* test power set at 0.8. The inclusion criteria consisted of noncomplicated total knee replacement surgery because of primary knee osteoarthritis and written consent to participate in the study. Exclusion criteria consisted of the following: complicated surgery, revision arthroplasty, total knee replacement because of other causes (secondary arthritis, tumour, trauma, etc.), current musculoskeletal complaints other than those related to the operated joint, other balance problems (due to neurological or heart diseases, vertigo, etc.), and refusal to participate in this study. Patients meeting the inclusion criteria were divided into two groups: a study group (VRG) and a control group (CG). The characteristics of the groups are shown in Table 1. The anthropometric parameters did not significantly differ between groups.

### 2.2. Gait Assessment

Gait was assessed using a Zebris FDM-T instrumented treadmill (Zebris Medical GmbH, Isny, Germany). Each patient walked at least 3 min on the treadmill before testing to become accustomed to this type of gait. Patients were instructed to look ahead and walk naturally during the test. Gait was assessed during 30 s of barefoot walking at a self-selected speed. All data were collected with a frequency of 120 Hz. The sensor resolution was 1.4 sensels/cm^2^. Two groups of parameters were collected during measurement: 16 parameters describing the force and foot plantar pressure and 20 spatiotemporal parameters. The first group of parameters included: left and right forefoot force (N), left and right backfoot force (N), left and right maximum forefoot force (N), left and right maximum midfoot force (N), left and right maximum heel force (N), left and right maximum forefoot pressure (N/cm^2^), left and right maximum midfoot pressure (N/cm^2^), left and right maximum heel pressure (N/cm^2^). The spatiotemporal parameters included the following: left and right step length (cm), stride length (cm), step width (cm), left and right stance phase (%), left and right loading response (%), left and right single limb support (%), left and right preswing (%), left and right swing phase (%), double support phase (%), left and right step time (s), stride time (s), cadence (step/min), and velocity (km/h).

For parameters assessed separately for the right (*R*) and left (*L*) lower limbs, symmetry indices were calculated according to the following formula [24]:

(1) $$SI=\frac{\left|X_{L}-X_{R}\right|}{0.5\cdot \left(X_{L}+X_{R}\right)}\cdot 100\%,$$

The *SI* factor is a method used to assess the percentage difference between parameters for both lower limbs during squats. An *SI* value of 0 indicates full symmetry, whereas *SI* ≥ 100% indicates asymmetry [24]. According to the assumption, the lower the *SI* values, the higher the symmetry.

### 2.3. Procedures

All patients who qualified for the study started a standard 4-week protocol of stationary rehabilitation involving five rehabilitation sessions per week immediately after the first assessment, each lasting approximately 4 h. Rehabilitation procedures were performed by two experienced physiotherapists. Physiotherapy treatment included the following: individual exercises (with a focus on increasing the range of motion of the operated joint, muscle stretching, and strengthening), continuous passive motion exercises, balance and gait training (without a computer or other electronic devices), kinesiology taping applications for oedema, classical massage, manual therapy (mainly soft tissue, patellar, and scar mobilization), and physical treatment (cryotherapy in the operated area, laser therapy for scarring, and low-frequency magnetic field therapy).

The study group additionally received 12 sessions (3 sessions per week) of nonimmersive virtual reality games on the Virtual Balance Clinic (VBC) prototype system (VBC-Project Consortium, Warsaw, Poland). The VBC system consists of two devices: (1) a balance plate (allowing to measure the displacement of the center of pressure (CoP) in real time) and (2) a “Kinect 2” camera (used to track body movements). Accordingly, VBC software allows the quantification of each movement performed during exercises as “correct”, “false”, or “partly correct”. The VBC system offers a choice of nine games in nonimmersive VR (Table 2). The VR games were applied concurrently with other treatments. Each exergaming session lasted 30 min and included three different games. Each patient played all games for the same length of time during the rehabilitation period. The level of difficulty was adjusted individually for each patient by a physiotherapist supervising the exercises. All patients completed their rehabilitation protocol. Each patient was assessed twice: before and after the 4-week rehabilitation.

### 2.4. Statistical Analysis

Statistical analysis was performed using Statistica v. 13.1 (TIBCO Software, Inc., Palo Alto, CA, USA), and the cut-off *p*-value was set to 0.05. Two groups of parameters, listed in Section 2.2., were used for the analysis.

The normality of the distributions of the abovementioned parameters was assessed using the Shapiro–Wilk test. Within groups CG and VRG, the effects of rehabilitation on parameter behaviour were examined using the Wilcoxon paired rank test. The t-test for independent groups was used if the variables were normally distributed in both groups. Using the Mann–Whitney U test (or a *t* test in case of normal distribution in both groups), the VRG and CG were then compared for all parameters assessed before and after rehabilitation. The percentage by which the parameter values increased or decreased after rehabilitation was also calculated. For parametric tests, the effect size was assessed using Cohen’s D, whereas the standardized z test statistic was used for nonparametric tests as follows: $r=\frac{\left|z\right|}{\sqrt{n}}$. Small effect sizes are for D = 0.2; D = 0.5 indicates a moderate effect size, and D = 0.8 indicated a large effect size. For nonparametric tests, the effect size ranges are *r* < 0.3 for a small effect size, 0.3 < *r* < 0.5 for a moderate effect size, and *r* > 0.5 indicates a large effect size.

## 3. Results

### 3.1. Comparison between the Study and Control Groups

Only five parameters were significantly different when comparing the VRG and CG for all parameters assessed before and after rehabilitation (Table 3). Before rehabilitation, only two parameters had significantly lower values in the study group (VRG) than in the control group (CG). After completing the rehabilitation program, differences were only observed in symmetry indices. Remarkably, the magnitude of the effect size was low (less than 0.3) for all studied parameters except for the SI_Loading response after rehabilitation, where the value of the effect size was in the lower range of the moderate effect.

### 3.2. Impact of Rehabilitation on the VRG and Control Groups

When analysing the effects of rehabilitation with virtual reality games, the values of 12 of 14 parameters evaluating the foot force and pressure significantly increased. The values of these parameters also increased in the control group, but the increase was significant for only six parameters. The most significant improvement was for symmetry indices. The value of SI_forefoot force decreased by 26.67% and 29.41% in the VRG and control groups, respectively. For the SI_Maximum forefoot force, the symmetry values improved by 25% for the VRG and 21.05% for the CG. Detailed values of the parameters that significantly changed are shown in Table 4 and Table 5.

Among the spatiotemporal parameters, the most significant improvement was in the symmetry indices for both SI_Loading response and SI_Pre-Swing, where values decreased by 54.55% and 140% in the VRG and C groups, respectively. Gait speed was another parameter for which significant changes were observed. It increased by 31.25% and 44% in the VRG and CG, respectively. The values of the parameters that significantly changed are shown in Table 6 and Table 7.

## 4. Discussion

The aim of this study was to assess the effectiveness of additional exercises in virtual reality in improving spatiotemporal and pressure distribution gait parameters in patients after total knee arthroplasty. The values of spatiotemporal parameters, foot loading, and foot pressure, as well as parameters assessing symmetry improved in both: the group that underwent the traditional rehabilitation protocol (CG) and the group that had an additional 12 sessions of exercise in a virtual environment (VRG). This result was confirmed both in tests for two-group comparisons and calculated effect sizes. However, the extra exergaming sessions did not significantly improve rehabilitation outcomes, which was previously confirmed by Rutkowski et al. [25].

When analysing the group of spatiotemporal parameters, walking velocity increased significantly after rehabilitation (31.25% and 44%) in the VRG and CG, respectively. This increase resulted in a significant reduction in the stride time (10% in the VRG group vs. 14.63% in the CG) and step time of both legs (mean 10% vs. 14.63%). Moreover, the higher velocity resulted in the shortening of the double support phase (12.2% vs. 12.99%) and stance phases for both limbs (mean 3.36% vs. 3.54%). These results were probably influenced by a significantly shorter loading response (mean 12.26% vs. 12.85%) and preswing (mean 12.31% vs. 13.07%) phases. Notably, step length (18.49% vs. 24.11%) and stride length (18.38 vs. 24.08%) significantly increased in both groups. According to Studenski et al. [26], gait speed is associated with survival among the elderly and reflects health and functional statuses. A gait speed faster than 100 cm/s suggests healthier ageing, whereas gait speeds slower than 60 cm/s indicate a likelihood of poor health and function. Furthermore, the walking speed in individuals after rehabilitation remained low in this study, amounting to 46.5 cm/s in the VRG and 49.8 cm/s in the CG. However, step width (21.07% vs. 18.53%) was reduced, suggesting more confident movements of the subjects [27].

The increase in gait speed after rehabilitation resulted in higher foot loading values and higher foot pressure distribution during gait. These results corroborate studies by Taylor et al. [28], Burnfield et al. [29], and Segal et al. [30]. Notably, the high forces and high-pressure distributions were placed in the heel and forefoot in both groups, which is related to the short phases of the loading response and preswing. This finding was confirmed by Jasiewicz et al. [31]. The above-described changes decreased the values of the symmetry coefficients, which approached zero, indicating an improvement in the symmetry of foot loading, foot pressure distribution, and spatiotemporal parameters.

The results of this study are consistent with our previously published results that showed no significant advantage of rehabilitation with additional VR training over standard rehabilitation in terms of balance and postural control in the same group of patients [11]. Gianola et al. [5] also reported no superiority of rehabilitation with VR in terms of pain relief, drug assumptions, and other functional outcomes. However, they observed some benefits in global proprioception in patients after TKR. Similar effects of rehabilitation protocols with and without VR were also reported by Gumaa and Rehan [16], Byra and Czernicki [22], and Blasco et al. [21]. Nevertheless, later reviews published by Peng et al. [23] and Gazendam et al. [17] showed some advantages of adding exergaming in VR to standard rehabilitation protocols. Peng et al. [23] found that VR-based rehabilitation reduced pain and improved function but had no significant effects on postural or balance control. Gazendam et al. [17] concluded that VR-based rehabilitation for patients undergoing TKR may be beneficial for some patients. Yoon and Son [32] confirmed significantly better balance in the group of patients after TKR who performed balance training with full immersion VR training. The patients in our study were trained in nonimmersive virtual reality, which can also influence the results. To date, no studies compared immersive and nonimmersive VR in patients after TKR.

Visual biofeedback, which allows the patient to automatically correct posture and movement to achieve the goal of the game, may be a beneficial factor of exergaming in VR. Cheung et al. [33] reported that biofeedback improved gait parameters in the gait retraining group in patients with knee OA. Christiansen et al. [8] confirmed this result; they obtained improved knee extension functions during gait in a group of patients after TKR who received biofeedback training to promote surgical limb loading. Some games used in our study were developed to retrain particular phases or types of gait, such as gait initiation (Fruits), single leg support (Frog, Football player, and Bicycle ride), and functional stepping (Bicycle ride and Frog). Other games (Boat, Colours, and Donkey) were included for balance training, which is also important in complex gait rehabilitation, but probably does not improve particular gait parameters.

An important factor that probably allows the patient to benefit from exergaming in VR is the level of difficulty and game scenario, which allows patients to maintain high concentration and motivation levels. Lee et al. [34] showed that the balance between task difficulty and personal skill level is a necessary prerequisite for immersion among patients after different knee surgeries (but not TKR). Additionally, Belchior et al. [35] suggested that older adults’ engagement during VR-based games is better when the level of difficulty can be adjusted to their skill levels. In our study, the difficulty level of each game was adjusted by the physiotherapist, which allowed us to maintain a balance between the patient’s effort and game results. However, patient motivation or satisfaction was not assessed in this study.

The patients included in our study started rehabilitation in the second week after surgery, and physical treatments lasted four weeks. For patients after TKR, this timepoint is an early period of rehabilitation, in which the main goals include tissue healing, increasing the range of motion, and restoring the basic function of the operated joint. Therefore, this period may be too early for reaching good results in advanced motor skills, such as balance or gait. This finding corroborates with that of Gazendam et al. [17] who showed that VR-based rehabilitation resulted in significantly better patient-reported outcome scores at 3 and 6 months postoperatively. However, the outcome in our study was based on objective measures and not patient-reported scores, as in many other studies [23].

### 4.1. Strengths and Limitations of the Study

The strength of this study is VR intervention, including a set of games dedicated towards improving balance and gait in nonhealthy people. Most studies are based on commercial solutions, such as Xbox Kinect or Nintendo Wii platform, which are not designed for balance or gait training, particularly in patients with musculoskeletal impairments [13,15,21,23,36]. Additionally, the intervention protocol (time and frequency of VR-sets) in this study was based on standards described by Juras et al. [37]. Another advantage is an objective measurement of the intervention’s effectiveness that was not dependent on the patient-reported outcome.

The limitations of this study include the lack of a healthy control group, which could allow studying how the individuals regained their normal gait parameters after TKR. The number of patients qualified for each group also differed (38 in the VRG and 21 in the CG). More patients (40 in each group) had been planned to be included in the study, but the project was completed at the end of 2019, and funds to include additional patients in the study group were not available. Second, the COVID-19 pandemic did not allow us to study more people, and qualifying patients for 4 weeks of rehabilitation was no longer possible due to limitations from the National Health Service. The use of exergaming sessions could also influence patients’ motivation, the intake of painkillers, the functioning of the neuromuscular system, or other factors that were not assessed in this study. The results could also be influenced by psychological complaints, that were not assessed.

Evaluating middle- and long-term outcomes after surgery in both groups of patients would be interesting for future studies. The 4-week rehabilitation was likely too short a period to achieve significantly better results in the VR group, and such benefits may become apparent over time. Future studies should also assess psychological complaints, patients’ motivation and pain (in the aspects of pain level, intensity, and painkillers’ intake). These aspects could influence the results or be influenced by therapeutic interventions.

### 4.2. Implications of Using Nonimmersive Virtual Reality in Clinical Practice

The incorporation of nonimmersive virtual reality games into everyday clinical practice requires more evidence. This study showed no clear advantages of adding dedicated VR games to the rehabilitation protocol for patients after total knee arthroplasty shortly after surgery. Virtual reality allows patients to train with biofeedback, receive some gratification (points in a game), and increase motivation and exercise without the constant supervision of a physiotherapist [38]. For elderly patients, nonimmersive VR is easier to work with than immersive VR, especially at the beginning. Therefore, nonimmersive VR can be safer for patients after experiencing lower limb surgery. These potential advantages can all improve rehabilitation results, although nonimmersive VR may be best suited for the later stages of rehabilitation from TKR.

## 5. Conclusions

Additional exercises in VR do not significantly improve pressure and spatiotemporal gait parameters compared with standard rehabilitation alone. Nevertheless, the improvement of gait, especially its symmetry, is significant within the first six weeks after surgery. Future studies that evaluate middle- and long-term effects and incorporate VR interventions into later stages of rehabilitation are warranted.

## Figures and Tables

**Table 1:** Characteristics of the participants (mean ± SD).

Group	Gender	Age (Years)	Body Mass (kg)	Body Height (cm)	Body Mass Index Bmi (kg/m^2^)
Study group (VRG) (*n* = 38)	26 females 12 males	68.6 ± 5.1	84.8 ± 14.2	164.9 ± 9.8	31.1 ± 3.4
Control group (CG) (*n* = 21)	14 females 7 males	68.4 ± 7.7	86.5 ± 17.7	167.5 ± 13.1	30.7 ± 4.4

SD—standard deviation, VRG—virtual reality group, CG—control group

**Table 2:** Virtual Balance Clinic games’ types, adapted from Ref. [10], Gait Posture 2021, on the basis of the International Association of Scientific, Technical & Medical Publishers (STM) Permissions Guidelines.

Game’s Name	Task Type	Task Description	Motor Ability	Progression Possibilities
Bicycle ride	Alternating steps without going forward with predetermined speed and frequency	Controlling the virtual avatar to ride on a bicycle at a given pace between two other cyclists	Leg coordination Single limb support Functional stepping	Frequency Height of leg lift
Boat	Leaning in the frontal plane, reaching toward a target at different directions, heights and depths	Standing on the balance board. Controlling the virtual boat to avoid obstacles by leaning body to the specified extent, maintaining this posture and reaching with the avatars’ hand toward a target to pick it up	Weight shifting Challenging limits of stability Functional transitions Arm coordination	Reaching length Number of obstacles and targets Range of weight distribution Speed Time of the training session
Colours	Leaning and maintaining the patient’s center of pressure (COP) in a given anterolateral direction. Remembering and repeating sequences of random directions	Standing on the balance board. Controlling the virtual point to reach and maintain the patient’s COP in a given colour by leaning in different directions	Weight shifting Training memory Advanced motor planning	Range of lean Number of remembered colour sequences (3–5)
Donkey	Trunk rotation with hands outstretched at shoulder height in a single leg forward standing position	Controlling the virtual avatar to reach a target and avoid obstacles while riding on a donkey by rotating the trunk	Trunk rotation	Number of obstacles Speed and targets
Football player	Random alternating front kicks	Controlling the virtual avatar to kick randomly approaching balls into the goal (from two different directions) by assigned leg (yellow ball-right leg, red ball-left leg; black-and-white ball-arbitrary leg; black ball-do not kick)	Single limb support Leg coordination Quick change of strategy Movement adaptation	Frequency of approaching balls Dual tasks Height of leg lift
Frog	Random alternating steps in 4 directions (sideways, forward, backward)	Controlling the virtual froglet to jump on the given leaf to catch the lightning bug by taking steps (with shifting body weight on the “active” leg) in the given direction	Functional stepping Leg coordination Single limb support Quick change of strategy	Step length Frequency of given step direction Time for movement execution
Fruits	Step initiation	Standing on the balance board. Controlling the robot’s arm in the single-leg forward standing position by shifting weight from one leg to another (initial position-grabbing the fruit, forward movement-lowering the fruit on the production line)	Weight shifting Gait initiation	Range of movement

**Table 3:** Results (mean ± SD) of the Mann–Whitney U test (*p* < 0.05) for comparison between the VRG and control groups.

Parameters	VRG	CG	*p*-Value	Effect Size
Before Rehabilitation				
SI_Maximum heel force (N)	0.30 ± 0.29	0.14 ± 0.17	0.0195	0.3040
Maximum right heel pressure (N/cm^2^)	18 ± 7.44	22 ± 8.28	0.0289	0.2844
After Rehabilitation				
SI_Loading response (%)	0.11 ± 0.10	0.05 ± 0.04	0.0121	0.3266
SI_Pre-Swing (%)	0.11 ± 0.10	0.05 ± 0.05	0.0195	0.3040
SI_Maximum heel force (N)	0.28 ± 0.29	0.12 ± 0.11	0.0283	0.2854

VRG—virtual reality group, CG—control group.

**Table 4:** Results (mean ± SD) of Wilcoxon test/*t*-test (*p* < 0.05) and effect size for comparison before and after rehabilitation within the VRG group for force and pressure parameters.

Parameters	Before	After	The Percentage of Increase↑/Decrease↓	*p*-Value	Effect Size
L Forefoot force (N)	531.6 ± 135.5 *	570.56 ± 159.5	7.33% ↑	0.0665	0.2388
R Forefoot force (N)	520.1 ± 123.01	563.62 ± 151.8	8.37% ↑	0.0687	0.3150
SI_Forefoot force (N)	0.19 ± 0.16 *	0.15 ± 0.16	26.67% ↓	*0.0445*	0.2614
L Backfoot force (N)	479.83 ± 122.3	516.77 ± 121.2	7.7% ↑	*0.0109*	0.3033
R Backfoot force (N)	448.47 ± 133.9	503.37 ± 133.4	12.24% ↑	*0.0013*	0.4106
Maximum L Forefoot force (N)	482.8 ± 127.4	526.16 ± 152.7	8.98% ↑	*0.0241*	0.3083
Maximum R Forefoot force (N)	468.25 ± 117.5	518.1 ± 148.3	10.65% ↑	*0.0394*	0.3725
SI_Maximum force Forefoot (N)	0.2 ± 0.16	0.16 ± 0.17	25% ↓	*0.0182*	0.2367
Maximum L Heel force (N)	386.95 ± 122.7	424.5 ± 127.1	9.72% ↑	*0.0101*	0.3011
Maximum R Heel force (N)	352.83 ± 117.2	408.67 ± 128.4	15.83% ↑	*0.0012*	0.4541
Maximum L Forefoot pressure (N/cm^2^)	23.55 ± 9.37	26.7 ± 11.04	13.38% ↑	*0.0027*	0.3084
Maximum R Forefoot pressure (N/cm^2^)	22.62 ± 9.62 *	25.93 ± 9.13	14.63% ↑	*0.0189*	0.3054
Maximum L Midfoot pressure (N/cm^2^)	12.53 ± 3.72	14.13 ± 4.48	12.77% ↑	*0.0074*	0.3873
Maximum R Midfoot pressure (N/cm^2^)	12.82 ± 3.86 *	15.1 ± 6.13 *	17.78% ↑	*0.0007*	0.4401
Maximum L Heel pressure (N/cm^2^)	19.45 ± 6.41	22.66 ± 8	16.5% ↑	*0.0008*	0.4433
Maximum R Heel pressure (N/cm^2^)	18.04 ± 7.44 *	21.17 ± 7.77	17.35% ↑	*0.0028*	0.3879

L—left lower limb, R—right lower limb, ↑/↓—the percentage of increase/decrease in parameter values in a given group after rehabilitation; significant *p*-values are indicated in italics; * indicates parameters with distributions different than normal.

**Table 5:** Results (mean ± SD) of Wilcoxon test/*t*-test (*p* < 0.05) and effect size for comparison before and after rehabilitation within control group for force and pressure parameters.

Parameters	Before	After	The Percentage of Increase↑/Decrease↓	*p*-Value	Effect Size
L Forefoot force (N)	512.3 ± 199.9 *	601.2 ± 230.9	17.35% ↑	*0.0041*	0.3733
R Forefoot force (N)	522.4 ± 231.9	610.2 ± 244.6	16.81% ↑	*0.0051*	0.3684
SI_Forefoot force (N)	0.22 ± 0.19 *	0.17 ± 0.19 *	29.41% ↓	0.3051	0.1334
L Backfoot force (N)	496.09 ± 108.1	514.76 ± 117.0	3.76% ↑	0.2586	0.1657
R Backfoot force (N)	499.71 ± 134.2	524.9 ± 135.43	5.04% ↑	0.1924	0.1868
Maximum L Forefoot force (N)	468.8 ± 191.9	562.3 ± 232.1	19.94% ↑	*0.0051*	0.4389
Maximum R Forefoot force (N)	481.3 ± 219.6	576.3 ± 238.9	19.75% ↑	*0.0029*	0.4140
SI_Maximum force Forefoot (N)	0.23 ± 0.19	0.19 ± 0.24 *	21.05% ↓	0.1808	0.1742
Maximum L Heel force (N)	411.23 ± 104.1	433.62 ± 112.1	5.44% ↑	0.1808	0.2068
Maximum R Heel force (N)	408.13 ± 135.8	438.39 ± 112.0	7.41% ↑	0.1924	0.1195
Maximum L Forefoot pressure (N/cm^2^)	22.63 ± 8.21	28.03 ±11.32	23.86% ↑	*0.0106*	0.5459
Maximum R Forefoot pressure (N/cm^2^)	23.58 ± 10.55 *	30.8 ± 12.67	30.62% ↑	*0.0007*	0.4411
Maximum L Midfoot pressure (N/cm^2^)	13.02 ± 3.32	14.125 ± 3.41	8.49% ↑	0.0734	0.3293
Maximum R Midfoot pressure (N/cm^2^)	15.35 ± 11.89 *	15.86 ± 10.90	3.32% ↑	0.1219	0.2013
Maximum L Heel pressure (N/cm^2^)	22.01 ± 6.83	23.55 ± 7.62	7% ↑	0.1396	0.2128
Maximum R Heel pressure (N/cm^2^)	22.15 ± 8.28	23.85 ± 5.72	7.67% ↑	0.2442	0.1515

L—left lower limb; R—right lower limb; ↑/↓—the percentage of increase/decrease in parameter values in a given group after rehabilitation; significant *p*-values are indicated in italic; * indicates parameters with distributions different than normal.

**Table 6:** Results (mean ± SD) of Wilcoxon test/*t*-test (*p* < 0.05) and effect size for comparison before and after rehabilitation within the VRG group for spatiotemporal parameters.

Parameters	Before	After	The Percentage of Increase↑/Decrease↓	*p*-Value	Effect Size
L Step length (cm)	30 ± 8.45	36.77 ± 8.07	22.57% ↑	*0.0001*	0.3959
R Step length (cm)	31.27 ± 7.22	35.78 ± 8.08	14.42% ↑	*0.0004*	0.4559
Stride length (cm)	61.27 ± 15.01	72.54 ± 15.46	18.38% ↑	*0.0001*	0.7399
Step width (cm)	14.25 ± 3.53	11.77 ± 4.07	21.07% ↓	*0.0001*	0.6500
L Stance phase (%)	71.97 ± 3.54	68.96 ± 3.33	4.36% ↓	*0.0002*	0.8782
R Stance phase (%)	71.1 ± 3.35	69.46 ± 3.12	2.36% ↓	*0.0207*	0.5076
L Loading response (%)	20.59 ± 2.97	18.94 ± 2.77	8.71% ↓	*0.0022*	0.5737
R Loading response (%)	22.47 ± 3.92 *	19.4 ± 3.11	15.82% ↓	*0.0003*	0.3914
SI_Loading response	0.17 ± 0.1	0.11 ± 0.1	54.55% ↓	*0.0151*	0.3162
L Single limb support (%)	28.93 ± 3.37	30.57 ± 3.08	5.67% ↑	*0.0170*	0.5074
R Single limb support (%)	28.02 ± 3.63	31.09 ± 3.32	10.96% ↑	*0.0002*	0.8845
L Pre-Swing (%)	22.5 ± 3.91 *	19.41 ± 3.11	15.92% ↓	*0.0003*	0.4230
R Pre-Swing (%)	20.61 ± 2.92	18.96 ± 2.79	8.7% ↓	*0.0022*	0.5793
SI_Pre-Swing	0.17 ± 0.1	0.11 ± 0.1	54.55% ↓	*0.0177*	0.3086
L Swing phase (%)	28.03 ± 3.54	31.04 ± 3.33	10.74% ↑	*0.0002*	0.8782
R Swing phase (%)	28.9 ± 3.35	30.54 ± 3.12	5.67% ↑	*0.0207*	0.5076
Double stance phase (%)	43.05 ± 5.67	38.37 ± 5.18	12.2% ↓	*0.0005*	0.8622
L Step time (s)	0.89 ± 0.16	0.81 ± 0.15 *	9.88% ↓	*0.0016*	0.4948
R Step time (s)	0.87 ± 0.16	0.79 ± 0.16 *	10.13% ↓	*0.0002*	0.4896
Stride time (s)	1.76 ± 0.31	1.6 ± 0.31 *	10% ↓	*0.0013*	0.4181
Cadence (steps/min)	70.71 ±11.98	77.7 ± 14.34	9.97% ↑	*0.0011*	0.5332
Velocity (km/h)	1.28 ± 0.33 *	1.68 ± 0.42	31.25% ↑	*0.0001*	0.5418

L—left lower limb; R—right lower limb; ↑/↓—the percentage of increase/decrease in parameter values in a given group after rehabilitation; significant *p*-values are indicated in italic; * indicates parameters with distributions different than normal.

**Table 7:** Results (mean ± SD) of Wilcoxon test/*t*-test (*p* < 0.05) and effect size for comparison before and after rehabilitation within the control group for spatiotemporal parameters.

Parameters	Before	After	The Percentage of Increase↑/Decrease↓	*p*-Value	Effect Size
L Step length (cm)	31.5 ± 9.14	40.04 ± 23.71	27.11% ↑	*0.0023*	0.3959
R Step length (cm)	31.69 ± 8.85	38.38 ± 4.34	21.11% ↑	*0.0142*	0.3190
Stride length (cm)	63.2 ± 16.19	78.42 ± 3.2	24.08% ↑	*0.0020*	0.7498
Step width (cm)	13.5 ± 3.97	11.39 ± 4.07	18.53% ↓	*0.0016*	0.5060
L Stance phase (%)	70.64 ± 4.34	68 ± 3.53	3.88% ↓	*0.0011*	0.6926
R Stance phase (%)	70.99 ± 3.58	68.78 ± 3.43	3.21% ↓	*0.0051*	0.5781
L Loading response (%)	20.59 ± 3.44	18.37 ± 0.04	12.08% ↓	*0.0078*	0.6365
R Loading response (%)	21.02 ± 4.5 *	18.5 ± 4.1	13.62% ↓	*0.0026*	0.3914
SI_Loading response	0.12 ± 0.12 *	0.05 ± 3.37 *	140% ↓	*0.0041*	0.3733
L Single limb support (%)	29.05 ± 3.56	31.24 ± 3.32	7.54% ↑	*0.0057*	0.5709
R Single limb support (%)	29.36 ± 4.35	31.91 ± 3.58	8.69% ↑	*0.0016*	0.6354
L Pre-Swing (%)	21.02 ± 4.43 *	18.42 ± 0.05	14.12% ↓	*0.0014*	0.4140
R Pre-Swing (%)	20.58 ± 3.37	18.37 ± 3.2	12.03% ↓	*0.0096*	0.6354
SI_Pre-Swing	0.12 ± 0.12 *	0.05 ± 4.07 *	140% ↓	*0.0096*	0.3371
L Swing phase (%)	29.36 ± 4.34	32 ± 6.8	8.99% ↑	*0.0011*	0.6926
R Swing phase (%)	29.01 ± 3.58	31.22 ± 0.22	7.62% ↑	*0.0051*	0.5781
Double stance phase (%)	41.58 ± 6.91	36.8 ± 0.2	12.99% ↓	*0.0013*	0.6965
L Step time (s)	0.95 ± 0.23	0.83 ± 15.4 *	14.46% ↓	*0.0172*	0.5103
R Step time (s)	0.93 ± 0.23	0.81 ± 0.42 *	14.81% ↓	*0.0051*	0.5472
Stride time (s)	1.88 ± 0.45	1.64 ± 0.72 *	14.63% ↓	*0.0078*	0.3461
Cadence (steps/min)	67.91 ± 16.33	76.72 ± 29.53	12.97% ↑	*0.0142*	0.5554
Velocity (km/h)	1.25 ± 0.35 *	1.8 ± 0.32	44% ↑	*0.0009*	0.4321

L—left lower limb; R—right lower limb; ↑/↓—the percentage of increase/decrease in parameter values in a given group after rehabilitation; significant *p*-values are indicated in italic; * indicates parameters with distributions different than normal.

## Data Availability

The measurement data used to support the findings of this study are available from the corresponding author upon request.
